# Nutraceuticals in the Prevention of Neonatal Hypoxia–Ischemia: A Comprehensive Review of their Neuroprotective Properties, Mechanisms of Action and Future Directions

**DOI:** 10.3390/ijms22052524

**Published:** 2021-03-03

**Authors:** Marta Reyes-Corral, Noelia Sola-Idígora, Rocío de la Puerta, Joan Montaner, Patricia Ybot-González

**Affiliations:** 1 Neurodevelopment Research Group, Institute of Biomedicine of Seville, IBIS/HUVR/CSIC/US, 41013 Seville, Spain; mreyesc-ibis@us.es (M.R.-C.); nosoid95@gmail.com (N.S.-I.); pybot-ibis@us.es (P.Y.-G.); 2 Department of Pharmacology, Faculty of Pharmacy, University of Seville, 41012 Seville, Spain; puerta@us.es; 3 Neurovascular Research Lab, Institute of Biomedicine of Seville, IBIS/HUVR/CSIC/US, 41013 Seville, Spain; 4 Department of Neurology and Neurophysiology, Hospital Universitario Virgen Macarena, 41009 Seville, Spain

**Keywords:** neonatal hypoxia–ischemia, nutraceuticals, natural products, neuroprotection, prevention, maternal supplementation, polyphenols, omega-3 fatty acids, vitamins, plant-derived compounds

## Abstract

Neonatal hypoxia–ischemia (HI) is a brain injury caused by oxygen deprivation to the brain due to birth asphyxia or reduced cerebral blood perfusion, and it often leads to lifelong limiting sequelae such as cerebral palsy, seizures, or mental retardation. HI remains one of the leading causes of neonatal mortality and morbidity worldwide, and current therapies are limited. Hypothermia has been successful in reducing mortality and some disabilities, but it is only applied to a subset of newborns that meet strict inclusion criteria. Given the unpredictable nature of the obstetric complications that contribute to neonatal HI, prophylactic treatments that prevent, rather than rescue, HI brain injury are emerging as a therapeutic alternative. Nutraceuticals are natural compounds present in the diet or used as dietary supplements that have antioxidant, anti-inflammatory, or antiapoptotic properties. This review summarizes the preclinical in vivo studies, mostly conducted on rodent models, that have investigated the neuroprotective properties of nutraceuticals in preventing and reducing HI-induced brain damage and cognitive impairments. The natural products reviewed include polyphenols, omega-3 fatty acids, vitamins, plant-derived compounds (tanshinones, sulforaphane, and capsaicin), and endogenous compounds (melatonin, carnitine, creatine, and lactate). These nutraceuticals were administered before the damage occurred, either to the mothers as a dietary supplement during pregnancy and/or lactation or to the pups prior to HI induction. To date, very few of these nutritional interventions have been investigated in humans, but we refer to those that have been successful in reducing ischemic stroke in adults. Overall, there is a robust body of preclinical evidence that supports the neuroprotective properties of nutraceuticals, and these may represent a safe and inexpensive nutritional strategy for the prevention of neonatal HI encephalopathy.

## 1. Introduction

Brain damage during late pregnancy and childbirth, mainly represented by hypoxia-ischemia (HI) encephalopathy, is a major cause of neonatal mortality worldwide. Approximately 40% of newborns with HI do not survive the neonatal period, and those who survive may have severe neurological morbidities such as cerebral palsy, visual and hearing impairment, seizures, epilepsy, mental retardation, or learning and communication problems [1]. This pathology affects 1–3/1000 in term infants (after 37 weeks of gestation) and 7/1000 in growth-restricted and preterm infants (before 37 weeks of gestation), with this figure increasing to 10–20/1000 live births in low-income countries [2,3]. Neonatal HI is global cerebral damage caused by inadequate blood flow and oxygen delivery to the brain as a result of a hypoxic–ischemic event during the prenatal, intrapartum, or postnatal period, such as birth asphyxia or intrauterine ischemia. HI encephalopathy can strike in pregnancies that have been uneventful until the final moments, and the nature of the obstetric complications that contribute to perinatal asphyxia is difficult to predict [4]. HI encephalopathy shares many common pathophysiological features with perinatal ischemic stroke, a focal ischemic brain injury that typically occurs between 28 weeks of gestation and postnatal day 28 [5].

The pathological events of HI encephalopathy occur in two phases: primary energy failure and secondary energy failure. Primary energy failure occurs as a result of the initial reduction of cerebral blood flow, which leads to severe oxygen and glucose deprivation, affecting the normal ionic gradients within the neuronal cells. This depolarization results in an excessive release of glutamate, which causes excitotoxicity and initiates the ischemic cascade [6,7]. The consequent intracellular influx of calcium triggers apoptosis, autophagocytosis, and necrotic pathways [8]. The low levels of glucose and oxygen also induce mitochondrial dysfunction, which occurs within minutes after the insult, resulting in the depletion of ATP production and the overproduction of reactive oxidative species (ROS) [9]. The generation of free radicals causes oxidative stress, which is particularly harmful to the neonatal brain due to the low concentration of antioxidants and the high consumption of oxygen when transitioning from fetal to neonatal life [10]. Increased calcium triggers nitric oxide (NO) production by the nitric oxide synthase (NOS), leading to brain damage [11,12]. The activation of the immune response within minutes after the ischemia triggers a cascade of immune cells that includes microglia, dendritic cells, macrophages, and lymphocytes, as well as the release of proinflammatory cytokines such as tumor necrosis factor α (TNF-α) or several interleukins (e.g., IL-1β, IL-6) This results in the breakdown of the blood–brain barrier (BBB), which in turn favors the infiltration of immune cells into the cerebral parenchyma and can lead to edema and tissue deterioration [13]. Immune cells also release inducible nitric oxide synthase (iNOS) that contributes to the harmful effect of NO on cerebral ischemia [14]. Once blood flow is restored, there is a brief period of recovery known as the latent period, characterized by normal cerebral metabolism. The secondary energy failure phase occurs 6 to 48 h after the initial injury and can last for days. This phase appears to be related to oxidative stress, excitotoxicity, and inflammation and is characterized by seizures, renewed cytotoxic edema, release of excitotoxins, impaired cerebral oxidative energy metabolism, and, finally, neuronal cell death [6,7].

The intensity of each of the events in the ischemic cascade will have an impact on the sequelae left by the brain injury, the treatment and care of which require significant resources. Even after maximal care, there is often little improvement in the general capabilities of newborns, with long-term burdens on the family and the healthcare system. Current therapies are limited. The most widely used is hypothermia, delivered through either selective head or whole-body cooling of the infant at 33–36.5 °C for 48–72 h. Hypothermia has been associated with a significant reduction in death and improved outcome at 18 months follow-up [15], but 40–50% of infants treated with hypothermia still die or develop chronic neurological impairments [16]. Moreover, hypothermia is only applied to a subset of newborns that meet strict inclusion criteria; it has a small therapeutic window (up to 6 h after birth), and it is largely restricted to use in tertiary-level medical facilities [17]. This strategy is aimed at reducing the spread of damage by reducing cerebral metabolic demand and inhibiting key steps in the excito-oxidative cascade, but it cannot prevent the injury or reduce susceptibility [1]. Therefore, new neuroprotective strategies for reversing/preventing the sequelae of neonatal HI need to be designed to ideally cover a greater percentage of affected newborns.

The balance of the pathophysiological response after an ischemic brain injury is critical to recovery, and all of the pathophysiological aspects have been evaluated as possible targets for neuroprotective therapies (reviewed in [18]). Therefore, research into the factors that lead to improved recovery and plasticity in the face of those that exacerbate ischemic damage is an important area for future translational research. In the adult population, the administration of natural neuroprotective compounds before an insult has shown beneficial effects in minimizing the neuronal damage induced by ischemic stroke [19]. This approach, known as advanced neuroprotective strategy (ADNES), consists of neuroprotective diets that include bioactive components with antioxidant or anti-inflammatory properties. The use of dietary interventions as a method of increasing adherence to treatment has been shown to reduce the risk of stroke in the adult population by achieving an environment of reduced excitotoxicity [20,21].

Lessons learned from nature show us that the environment, condition, and/or genotype of the mother can modulate the phenotype of her offspring, in some cases reversing the developmental instructions conferred by the offspring’s genotype. This is what is known as the maternal effect, which gives plasticity to the phenotype of the offspring to adapt to different environmental situations [22]. Thus, an effective strategy for the prevention of developmental diseases would be to treat the mother during pregnancy to alter the fetal environment and, in turn, modulate the phenotype of the fetus. In this way, maternal folic acid supplementation has been shown to be effective in reducing the incidence of neural tube defects [23,24]. Maternal nutrition during pregnancy is a research topic of growing interest in the field of pediatric ischemia as it may have an impact on both the development of offspring as well as the provision of neuroprotection. Furthermore, we believe that to maximize the benefits of ADNES designed to prevent neonatal HI, the dietary interventions should be as healthy and natural as possible to promote pregnant women’s adherence to these diets.

Animal models are the first step in exploring the mechanisms that underlie disease and evaluating the safety and efficacy of treatments. Particularly in models of perinatal brain damage, the success of generating reliable models for human development will depend largely on obtaining similarities in the function and development of the central nervous system (CNS) between species. In both humans and rodents, CNS development is achieved in the postnatal age, and cross-comparisons of macroscopic neuroanatomy have shown similarities in the timing of neurogenesis, synaptogenesis, glycogenesis, maturation, myelination, as well as in age-dependent molecular and biochemical changes. The rodent brain at postnatal day 1 (P1)–P5 corresponds to 23–32 weeks of gestation in humans and is, therefore, suitable for studying lesions in preterm patients. On the other hand, the rodent brain at P7–P10 corresponds to 36–40 weeks of gestation in humans and is, therefore, suitable for studies of brain injury in term patients [25]. Although most studies of neonatal HI use rodent models, other species such as piglets [26], rabbits [27], sheep [28], and nonhuman primates [29] are also used.

Experimentally, one way to make animal models of HI encephalopathy comparable to those observed in humans is to induce lack of oxygen (hypoxia) and reduce blood perfusion in the brain (ischemia) over a significant period of time for damage to occur. The most commonly used method in the immature animal is the Rice–Vannucci method [30], based on the previous protocol described by Levine [31] for adult rodents. The Rice–Vannucci method consists of the unilateral ligation of the common carotid artery, followed by exposure to hypoxia using 8% O_2_; this is usually performed at P7 [30]. This model causes hypoperfusion on the ligated side of the brain, while the unlinked side serves as a control by being exposed to hypoxia only. The length of hypoxic exposure (typically between 45 min and 2.5 h) can lead to mild, moderate, or severe HI damage [32]. Another protocol used in rodents is the Wigglesworth model of fetal growth restriction. In this protocol, the uterine and/or ovarian vessels are ligated or occluded, uni- or bilaterally, to induce chronic placental insufficiency at embryonic day 19–20 (E19–E20), considering that gestation lasts 23 days [33]. This protocol is exclusive for the study of HI in preterm neonates. Moreover, other models of brain damage due to perinatal asphyxia can be achieved by exposing the pups to a mixture of asphyxiation gas that combines hypercapnia (20% CO_2_) and hypoxia (9% O_2_) [34] or by inducing intrauterine ischemia with a “delayed cesarean section”, a protocol developed by Bjelke et al. [35], in which the pregnant uterus is dissected at the end of the gestation period and placed in a pre-warmed saline bath before the fetuses are extracted. Finally, another model of perinatal brain injury caused by glutamate-induced excitotoxicity is achieved by injecting glutamate or its analog ibotenate into the brain at P5 [36,37]. As nearly all the investigations published to date have utilized rat and mouse models, this review will focus on those species but will also provide information on other species, especially humans, where such information is available.

The present review summarizes the preclinical in vivo studies and the few available human clinical studies in which nutritional interventions were applied as prophylaxis before the HI cerebral damage, either as a maternal dietary supplementation during pregnancy or to the offspring before HI induction. The interventions examined only included natural products and nutraceuticals, which may be attractive alternatives to traditional drugs as they have a low toxicity profile, are comparatively affordable, and are widely available [38,39,40]. The neuroprotective and preventive properties of polyphenols, omega-3 fatty acids, vitamins, and other plant-derived and endogenous compounds in the context of neonatal HI are summarized below.

## 2. Methods

**Figure 1:**
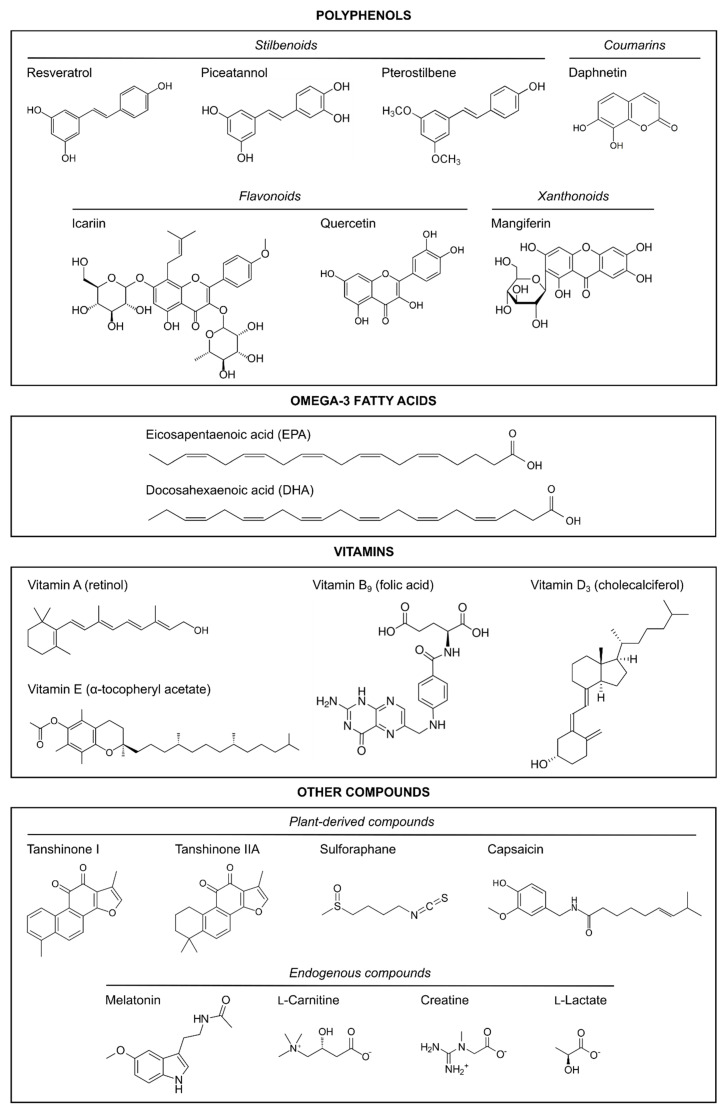
Chemical structures of the natural neuroprotective compounds reviewed here.

The articles reviewed herein were collated from the PubMed database, which was searched up to January 2021 with the following search, which excluded reviews and those articles not written in English: ((((((“hypoxia”[MeSH Terms] OR hypoxi*[Text Word] OR anoxi*[Text Word]) AND (“ischemia”[MeSH Terms] OR ischemi*[Text Word])) AND (((((((pregnancy) OR (gestational)) OR (maternal)) OR (prenatal)) OR (antenatal)) OR (neonatal)) OR (perinatal))) AND (((neuroprotect*) OR (prevent*)) OR (anti-inflammatory))) AND ((((((((((natural) OR (nutrient)) OR (nutrition*)) OR (nutraceutical)) OR (supplement*)) OR (diet*)) OR (vitamin)) OR (polyphenol)) OR (melatonin)) OR (polyunsaturated fatty acid))) AND ((((((((((((((human) OR (female)) OR (women)) OR (mother)) OR (animal model)) OR (rodent)) OR (murine)) OR (rat)) OR (mouse)) OR (mice)) OR (guinea pig)) OR (pig)) OR (sheep)) OR (rabbit))) AND ((((fetus) OR (newborn)) OR (neonate)) OR (pup)) AND (english[Filter]) NOT (“review”[Publication Type]). The search resulted in 256 articles, but the following were excluded: in vitro studies, ex vivo studies, and those in which the neuroprotective strategy was applied after the HI damage had occurred (i.e., as a post-treatment to pups or newborns). After reading the abstract, and, in some cases, the entire manuscript, a total of 49 studies fitted the inclusion criteria; only two of those were clinical studies conducted on humans [41,42]. The preclinical in vivo studies using natural products with neuroprotective properties in neonatal HI animal models are summarized in Table 1. The chemical structures of the natural compounds reviewed are shown in Figure 1.

## 3. Polyphenols

Polyphenols are a diverse group of plant-derived organic compounds characterized by having several hydroxyl groups on aromatic rings. These molecules are secondary metabolites that fulfill a very broad range of physiological roles in plants and are generally involved in defense against ultraviolet radiation or aggression by pathogens [91,92]. Polyphenols display numerous biological activities, and dietary polyphenols present in fruits and vegetables have shown beneficial effects in the treatment and prevention of several diseases, including cancer [93,94], neurodegenerative disorders [95,96], and diabetes [97]. Some polyphenols have been demonstrated to possess neuroprotective effects against brain injury; below, we summarize their properties in relation to neonatal HI.

### 3.1. Resveratrol

Resveratrol (3,5,4′-trihydroxystilbene) is a natural stilbene polyphenol found in many plant species and fruits such as grapes, peanuts, pomegranates, and some berries [98]. The most common dietary source of resveratrol is red wine, and this polyphenol is believed to be an important factor in the French Paradox: the observation that the French population has a very low incidence of cardiovascular disease despite a high-fat diet [99]. Resveratrol is one of the most extensively studied polyphenols, and it has anticarcinogenic, anti-inflammatory, and antioxidant properties [100,101]. However, its oral bioavailability is low (<1%) as it undergoes extensive metabolization in the intestine and liver [102]. Resveratrol can cross the placenta and reach fetal circulation [103], and its maternal consumption during pregnancy (at doses up to 750 mg/kg/day) is safe [104]. Several studies have examined the neuroprotective properties of resveratrol in the context of neonatal HI when injected intraperitoneally to pups prior to an HI insult [43,44,45,46] or when administered to the mothers as a dietary supplementation [47,48].

Using the Rice–Vannucci method in P7 mouse and rat pups, West et al. [43] showed that resveratrol at 20 mg/kg protected against tissue loss in the hippocampus and striatum and reduced apoptotic and necrotic cell death in a dose-dependent manner when injected intraperitoneally to mice pups 10 min or 24 h before HI [43]. The finding that resveratrol was neuroprotective even when given 24 h before the injury could either be due to its slow metabolism in mice [105] or to its ability to mimic the effects of preconditioning, as shown in a rat brain slice model of ischemia [106]. In rat pups, intraperitoneal injection of 20 mg/kg resveratrol 10 min before the hypoxic insult reduced caspase-3 activation [43]. Using the same resveratrol treatment modality and dose in P7 rat pups, Arteaga et al. [44] showed that resveratrol could ameliorate HI-induced morphological damage, reducing infarct volume, loss of myelination, and cell loss, especially in the cortex and the hippocampus. The same authors showed that this resveratrol pretreatment also reduced morphological damage and astrogliosis in the inferior colliculus and restored the auditory brainstem functional response that was affected by HI [44]. Resveratrol prevented HI-induced long-term cognitive impairments and functional damage in adult rats, evaluated at P90 [44]. The authors postulated that one of the mechanisms by which resveratrol protects against HI may be the maintenance of mitochondrial inner membrane integrity and transmembrane potential, as well as the reduction of ROS production [44]. Similarly, Gao et al. [46] showed that intraperitoneal administration of 20 or 40 mg/kg/day resveratrol for a week prior to HI induction in P14 rat pups resulted in a reduction of the infarct area and HI-induced cerebral edema. Resveratrol attenuated the HI inflammatory response, reducing the expression of proinflammatory cytokines IL-6, IL-1β, TNF-α, and NF-κΒ p65 subunit. Additionally, resveratrol reduced oxidative stress by enhancing the activities of the antioxidant enzymes glutathione peroxidase, catalase, and superoxide dismutase. The authors suggested that resveratrol activates the Nrf2/HO-1 (nuclear factor erythroid 2 related factor 2/heme oxygenase 1)-dependent signaling pathway to produce endogenous antioxidants that may contribute to its neuroprotective activity [46].

Besides the aforementioned studies, in which resveratrol was injected intraperitoneally to pups, Isac et al. [47] investigated maternal dietary supplementation with resveratrol (50 mg/kg/day) in neonatal rats at P6 using a modified version of the perinatal asphyxia model developed by Helmy et al. [89]. In this case, maternal dietary supplementation with resveratrol was administered from a period that initiated when female rats finished weaning, which continued while dams reached maturity for mating and during the whole pregnancy and the first week of lactation until the offspring reached P7. The authors assessed the hippocampal expression of neuroinflammation and neural injury markers TNFα, IL-1β, and S-100B, as well as the expression of several small noncoding microRNAs (miR124, miR132, miR134, miR15a, and miR146) involved in the epigenetic control of neuroinflammation, tolerance to asphyxia, apoptosis, angiogenesis, and neuronal maturation. They found that maternal supplementation with resveratrol could reduce the expression levels of TNFα, IL-1β, and S-100B, which increased secondary to perinatal asphyxia, similar to previously observed results [46]. Although the expression of some microRNAs was altered following perinatal asphyxia, resveratrol did not induce any significant changes in their expression. The authors suggested that resveratrol can reduce asphyxia-related neuroinflammation and neural injury [47]. Given the long period of resveratrol supplementation employed in this study, this polyphenol may have not only interfered with fetal development but also with maternal growth before gestation, something that was not investigated further by the authors.

Dumont et al. [48] also followed a maternal dietary supplementation regime, initiated during the last week of gestation and maintained until pups reached P9, in order to investigate the neuroprotective role of resveratrol in the context of moderate maternal alcohol consumption [48]. They used a nutritional dose of resveratrol of 0.15 mg/kg/day, which, based on the content of this polyphenol present in various raisins [107], would be equivalent to the consumption of about 22 g of grapes (30 grape berries) per day for a pregnant woman. Contrary to other findings [43,44,45,46,47], in this study, resveratrol only showed partial neuroprotection against HI. Resveratrol did not reduce HI-induced brain lesions, but it counteracted some deleterious sensorimotor defects and hippocampal-dependent long-term memory deficits induced by HI and alcohol consumption, respectively. Its hydroxylated analog piceatannol displayed higher neuroprotection than resveratrol [48] (see Section 3.2.1). The discrepancies in resveratrol’s neuroprotective properties can be related to the concentration of the polyphenol employed, since the concentration used by Dumont et al. [48] was up to two orders of magnitude lower than the concentrations used in other studies [43,44,45,46,47] (they were all conducted using the Rice–Vannucci HI model). Given the low oral bioavailability of resveratrol [102], the mode of administration (maternal supplementation vs. intraperitoneal injection to pups) may also interfere with resveratrol actions.

Finally, although the scope of this review are natural preventive agents that can protect against HI when administered before this damage occurs, it is interesting to mention that in the studies by Arteaga et al. [44] and West et al. [43], resveratrol did not exert neuroprotection when administered after the hypoxic damage. However, other studies have shown that resveratrol treatment after HI can ameliorate HI-induced brain damage [108,109] and behavioral deficits [108] in the Rice–Vannucci rat model. These discrepancies are likely due to the use of higher concentrations of resveratrol (20 mg/kg in [43,44] vs. 90 mg/kg in [108] and 100 mg/kg in [109]) since the neuroprotective effects of this polyphenol are dose-dependent in rats [46,110,111]. Hence, when administered as a therapeutic agent rather than a preventive agent, the damage produced by the deleterious HI cascade has already started, and the dose of resveratrol needed to revert the damage may be higher.

### 3.2. Resveratrol Derivatives: Piceatannol and Pterostilbene

#### 3.2.1. Piceatannol

Piceatannol (3,3′,4′,5-tetrahydroxystilbene) is a hydroxylated analog of resveratrol that occurs naturally in berries, grapes, passion fruit, and white tea, and it has anticancer and anti-inflammatory properties [112]. In humans, resveratrol can be metabolized to piceatannol by the cytochrome P450 enzyme CYP1B1 [113], which is overexpressed in a wide range of tumors but not in adjacent normal tissue [114]. Piceatannol has higher biological activity and metabolic stability [115] as well as stronger antioxidant activity [116] than resveratrol. Despite the accruing evidence indicating that resveratrol could play a neuroprotective role in the context of perinatal HI [44,45,46,47,108,109] (summarized in Section 3.1), its analog piceatannol has received limited attention. Two studies by Dumont et al. [48,49] evaluated maternal dietary supplementation with piceatannol as a preventive nutritional approach against HI. Using the Rice–Vannucci HI model, piceatannol was administered to the mothers in drinking water during the first week of lactation or the last week of gestation plus the first week of lactation in a dose equivalent to one passion fruit per day for a pregnant woman (0.15 mg/kg/day). In the short term, piceatannol reduced cerebral edema and decreased cell death 48 h post-HI, and it also improved early reflexes evaluated at P8–P12. The neuroprotective effects of piceatannol were still present in the longer term. In juvenile rats, maternal piceatannol supplementation reversed anatomical brain lesions, improved the spatial distribution of white matter fiber bundles, and counteracted sensorimotor deficits and long-term memory impairments, which were comparable to those of sham controls [49]. Additionally, piceatannol supplementation allowed pups to recover their sensorimotor and cognitive functions after a HI event in a context of moderate maternal alcohol consumption, whereas resveratrol only exerted partial neuroprotection when used at the same dose of 0.15 mg/kg/day [48]. The authors explained that the increased neuroprotective effect of piceatannol compared to resveratrol is conferred by the additional hydroxyl group that piceatannol possesses in its chemical structure. Moreover, they also suggested that, in addition to their antioxidant properties, piceatannol and resveratrol neuroprotection may be multimodal and implicate the regulation of brain metabolism. In short, Dumont et al. postulated that these polyphenols may prevent neuronal death and reduce brain damage which leads to motor and cognitive impairments by increasing glycolysis (and, therefore, lactate levels) in astrocytes, which could then spare glucose for its metabolic use through the pentose phosphate pathway in neurons and enhance their reduced glutathione levels [48,49].

#### 3.2.2. Pterostilbene

Pterostilbene (3,5-dimethoxy-4′-hydroxystilbene) is a naturally occurring dimethoxylated structural analog of resveratrol present in red sandalwood, grapevines, and blueberries, and it is the major phenolic component in some traditional Ayurvedic medicines [117,118]. Pterostilbene has shown anticancer, cardioprotective, and neuroprotective properties [117] and has better pharmacokinetic characteristics than its analog resveratrol due to the two methoxy groups of its structure, which make it more lipophilic, thus increasing its oral bioavailability (~12.5%) [119,120]. A study by Li et al. [50] analyzed the neuroprotective properties of pterostilbene in P7 rat pups subjected to the Rice–Vannucci protocol. Pterostilbene, injected intraperitoneally at 50 mg/kg 30 min prior to HI induction, decreased brain infarct volume and brain edema and improved both motor and working memory deficits secondary to HI. Additionally, pterostilbene pretreatment decreased the expression of proinflammatory cytokines IL-6, IL-1β, TNF-α, and NF-κΒ p65 subunit (as shown for resveratrol [46,47]) and reduced oxidative stress and apoptosis. Zinc protoporphyrin IX, an HO-1 inhibitor, was able to inhibit the pterostilbene-induced suppression of oxidative stress, programmed cell death, inflammation, and brain damage [50], indicating that pterostilbene pretreatment may prevent HI damage through the upregulation of HO-1, as suggested for its analog resveratrol [46].

### 3.3. Quercetin

Quercetin (3,3′,4′,5,7-pentahydroxyflavone) is a ubiquitous flavonol present in most plants, fruits, and vegetables. It can reach levels in the human diet as high as 3–38 mg/day [121], with some of the major dietary sources of quercetin being onions, broccolis, apples, leeks, kales, tea, and red wine [122,123]. Quercetin has shown anticancer, cardioprotective, and antioxidant activities [124,125,126], and numerous studies have endorsed quercetin in vivo and in vitro neuroprotective properties in the context of focal ischemia [127,128,129,130] or oxygen–glucose deprivation injury [130,131,132]. However, quercetin suffers from poor bioavailability as it is rapidly and extensively metabolized and excreted [124], and there are contradictory results on its ability to cross the BBB [133]. These pharmacokinetic properties are not favorable for its acute intravenous administration for the treatment of perinatal HI and, therefore, other administration strategies have been investigated, such as intragastric administration [52,134,135] or the use of liposomal preparations, which facilitate its permeability to the brain [128,136]. Regarding the latter, Blasina et al. [51] developed nanosomes of quercetin (i.e., nanometer-sized vesicles of phospholipid bilayers) that were administered intravenously to piglets that underwent bilateral transient carotid ligation, followed by 40 min hypoxia 48 h after birth. Quercetin nanosomes (10 mg/kg) improved electroencephalographic amplitude records, although no histopathological differences in brain lesions were observed compared to untreated controls. Piglets receiving nanosomes also stabilized blood pressure and recovered spontaneous breathing 8 h after HI and showed better suckling and walking capacity 3 days after HI [51].

Wu et al. [52] used the Rice–Vannucci model and administered quercetin intragastrically for a week prior to HI induction to P7 rats at a dose of 40 mg/kg/day. Twenty-four hours after HI, quercetin was shown to reduce cortical cell apoptosis by modulating the expression of proteins in the apoptotic pathway; it attenuated cortical cell microgliosis and astrogliosis, and it partially reversed the neuroinflammation induced by HI injury by reducing the expression of proinflammatory markers IL-6, IL-1β, and TNF-α (as shown for other polyphenols [46,47,50]). The authors showed that one of the mechanisms involved in quercetin neuroprotection was the suppression of the TLR4/NF-κB (Toll-like receptor 4/nuclear factor κB) signaling pathway-mediated neuroinflammatory response [52]. This is not the only report that has suggested quercetin modulates TLR4/NF-κB signaling to exert neuroprotection, as others have shown that quercetin suppresses the TLR4/NF-κB pathway and oxidative stress in vitro using an oxygen–glucose deprivation injury model in microglial cells [132].

Although not part of the scope of this review, it is worth noting that treatment with quercetin after an HI insult has also shown to be neuroprotective in vivo. Hence, intraperitoneal injection of 50 mg/kg quercetin for three consecutive days after HI induction to P7 mice reduced brain infarct volume and improved long-term motor and cognitive function [132]. Moreover, quercetin intragastric administration at 20 or 40 mg/kg/day for six weeks after HI insult to P3 rats resulted in a reduction of HI-induced cognitive deficits [134,135] and also improved remyelination by promoting the proliferation of oligodendrocyte progenitor cells and strengthening the survival of oligodendrocytes [135]. Based on the aforementioned observations of quercetin neuroprotective properties in the context of neonatal HI [51,52,132,134,135] and given that this flavonoid is abundantly present in the diet [121,122,123], it would be interesting to investigate its potential as a nutraceutical to evaluate whether maternal supplementation with quercetin could be an effective preventive strategy against neonatal HI.

### 3.4. Mangiferin

Mangiferin (C-glucopyranoside 1,3,6,7-tetrahydroxyxanthone) is a natural polyphenol from the xanthone family present in numerous plant species, and it is particularly abundant in the fruit peels, leaves, stem bark, and roots of the mango tree (*Mangifera indica* L.) [137,138]. Mangiferin has known antiangiogenic, anticancer, immunomodulatory, and antioxidant activity; the latter is related to the C-glucosyl linkage and the presence of multiple hydroxyl groups of its xanthonoid structure, which contribute to its free radical-scavenging activity [139,140]. Using a neonatal rat HI model, Xi et al. [53] showed that postnatal administration of mangiferin exerted neuroprotection in a dose-dependent manner. Mangiferin reduced brain infarct volume, improved histological changes in the hippocampus following HI damage, reduced neuronal cell death by regulating the expression of several proteins in the apoptotic cascade, and attenuated oxidative stress by reducing ROS and malondialdehyde levels [53]. Additionally, mangiferin potentiated the known neuroprotective activity of the anesthetic isoflurane [53,141,142,143]. The authors suggested that mangiferin-induced neuroprotection is related to the activation of the PI3K/Akt (phosphoinositide 3-kinase/protein kinase B) signaling pathway, which was downregulated following HI insult, and concluded that mangiferin is a promising therapeutic agent in the treatment of neonatal HI, administered alone or with isoflurane [53]. It remains to be determined whether the neuroprotection exerted by mangiferin translates to better sensorimotor and cognitive outcomes following HI.

### 3.5. Pomegranate Juice Polyphenols

Pomegranate juice has antioxidant, anti-inflammatory, antimicrobial, and antidiabetic activities, and it possesses a higher concentration of polyphenols than juices extracted from other fruits. Its polyphenolic composition includes tannins, anthocyanins, proanthocyanidins, flavonoids, and several phenolic acids [144,145]. Pomegranate polyphenols have shown neuroprotective and antioxidant properties against ischemia in vitro [146], in vivo [147,148], and in a randomized controlled trial in which pomegranate supplementation enhanced cognitive and functional recovery in stroke patients [149]. Loren et al. [54] showed that supplementing the maternal diet with pomegranate juice during the peripartum period (for 15 or 21 days) provided significant neuroprotection from HI injury to neonatal mice. Pomegranate juice protected against brain tissue loss in the hippocampus, cortex, and striatum in a dose-dependent manner (at 8, 16, and 32 µmol/day), and it reduced caspase-3 activation. These results were replicated in a later study by the same authors, which showed a similar reduction in apoptotic neuronal cell death after pomegranate juice supplementation [43]. Liquid chromatography-mass spectrometry showed that ellagic acid, a polyphenol found in pomegranate juice, was present in pup serum, confirming the maternal gastrointestinal absorption of pomegranate juice components and their passage across the mouse placenta from maternal serum to pup serum [54].

In humans, a randomized, placebo-controlled, double-blind pilot study with 77 participants investigated the impact of maternal pomegranate juice intake in pregnancies with intrauterine growth restriction [41], a significant complication of pregnancy defined as a pathological decrease in the rate of fetal growth [150]. Fetuses with intrauterine growth restriction often suffer long-term placental insufficiency, resulting in chronic hypoxia similar to acute perinatal HI injury [150]. Maternal intake of 8 oz. of pomegranate juice (equivalent to ~237 mL and >700 mg of gallic acid equivalent (GAE) polyphenols) for an average of 20 days until delivery did not significantly affect brain macrostructure, i.e., brain injury, metrics, or volume. However, pomegranate juice intake was associated with altered white matter organization in the corpus callosum and bilateral anterior and posterior limbs of the internal capsule, as well as enhanced functional connectivity within the visual network of the infant brain [41]. This is one of the few human studies that have examined the effects of a gestational dietary intervention in pregnant women at risk of having a baby affected by neonatal HI. The results from this pilot study suggest differences in brain structure and function following in utero exposure to pomegranate juice [41], which would require further investigation to establish its potential preventive effects against perinatal brain injury.

### 3.6. Grape Seed Proanthocyanidin Extract

Grape seed proanthocyanidin extract (GSPE) is a biological polyphenolic compound commonly used as a dietary supplement. GSPE contains flavanols that range in molecular weight from monomers (mainly catechin, epicatechin, and their gallate forms) to long-chain polymers primarily composed of dimeric and trimeric procyanidins [151]. Proanthocyanidins are also present in most fruits, especially in berries, as well as in flowers, nuts, bark, and seeds of various plants [151]. GSPE has antioxidant, anti-inflammatory, anticancer, antihyperglycemic, and cardioprotective properties [151]. It has been shown to be neuroprotective against ischemia–reperfusion brain injury in the adult brain of mice by attenuating oxidative stress and apoptosis and promoting angiogenesis [152]; it has also been shown to reduce HI-induced brain injury by suppressing lipid peroxidation when administered to neonatal rats after an HI insult [153,154]. Tu et al. [55] reported that GSPE pretreatment was neuroprotective against HI damage using the Rice–Vannucci model in P7 mice. Intraperitoneal administration of 30 mg/kg GSPE 20 min prior to HI damage reduced brain infarct volume and attenuated HI-induced neuronal apoptosis. GSPE pretreatment also potentiated functional recovery after injury and resulted in an improvement of neurobehavioral outcomes compared to untreated HI controls [55]. Another study in pregnant mice exposed to an acute intake of GSPE showed that the placenta seemed to act as a barrier for the transport of GSPE flavanols and their metabolites to the fetus. However, trace amounts of these compounds reached the fetus, suggesting that they could exert a biological effect on the offspring [155]. It would be, therefore, interesting to determine whether maternal administration of GSPE during pregnancy could be used as a neuroprotective dietary strategy for the prevention of neonatal HI, as shown for other polyphenols [43,47,48,49,54].

### 3.7. Other Polyphenols from Traditional Chinese Medicines: Icariin and Daphnetin

#### 3.7.1. Icariin

The flavonoid icariin is the main active component of the traditional Chinese medicinal herb *Epimedium grandiflorum* C. Morren, native to China, Japan, and Korea. Icariin is the 8-prenyl derivative of the flavonol kaempferol 3,7-O-diglucoside, and it has shown antiapoptotic, anti-inflammatory, and antioxidative properties in a wide variety of disorders, such as neurodegenerative disease, cardiovascular disease, and osteoporosis [156,157]. For instance, icariin attenuated neuronal damage following cerebral ischemia-reperfusion injury in rats [158] and oxygen–glucose deprivation/reperfusion injury in vitro [159] through the inhibition of inflammation and apoptosis, respectively. A study by Wang et al. [56] showed that intraperitoneal injection of 10 mg/kg icariin 20 min before HI induction in P7 mice resulted in a reduction of brain infarct volume as well as improved growth and functional recovery; the latter was evaluated at 1, 3, and 7 days after the HI insult. Icariin inhibited HI-induced apoptosis and activated the PI3K/Akt signaling pathway, which was reduced after HI injury. These findings suggest that icariin may play a neuroprotective role in neonatal HI via the activation of prosurvival signaling pathways and the inhibition of proapoptotic signaling pathways [56].

#### 3.7.2. Daphnetin

Daphnetin (7,8-dihydroxycoumarin) is a coumarin derivative extracted from several plants and shrubs of the genus *Daphne*, such as *D. giraldii*, *D. marginate*, and *D. odora*, and it is the major component of some traditional Chinese medicines used for the treatment of coagulation disorders and rheumatoid arthritis [160,161]. Daphnetin displays analgesic, anti-inflammatory, antimalarial, antimicrobial, and antioxidant properties [160,162,163,164,165]. A study by Du et al. [57] showed that intraperitoneal injection of 10 mg/kg of daphnetin 1 h prior to the HI insult to P7 rat pups resulted in a reduction of brain infarct volume, whereas daphnetin administered post-treatment 4 or 6 h after HI caused partial or no reduction in infarct volume, respectively. The same study showed that daphnetin reduced brain infarct volume and improved neurological deficits after ischemic injury using a middle cerebral artery occlusion mouse model [57], as shown by others [166]. Daphnetin also protected hippocampal neurons against glutamate-induced cell death by reducing oxidative stress in vitro [57], in agreement with another report showing that daphnetin attenuated oxidative stress and neuronal apoptosis after oxygen–glucose deprivation/reoxygenation injury in hippocampal cells through the activation of the Nrf2/HO-1 signaling pathway [167]. The latter observations suggest that the reduction of oxidative stress may be the mechanism by which daphnetin exerts its neuroprotection against neonatal HI. However, this mechanism and the putative effects of daphnetin in preventing cognitive impairments and functional damage secondary to HI remain to be investigated in vivo. Moreover, given that daphnetin is used for the treatment of coagulation disorders [160,161], its anticoagulant properties [168,169] and safety would need to be carefully evaluated in the context of neonatal HI.

## 4. Omega-3 Fatty Acids

Omega-3 (n-3) fatty acids are a family of long-chain polyunsaturated fatty acids (PUFAs) characterized by the presence in their chemical structure of a double bond three atoms away from the terminal methyl group. Several different omega-3 fatty acids exist, but the three main omega-3 fatty acids involved in human physiology are α-linolenic acid (ALA; C18:3n-3), eicosapentaenoic acid (EPA; C20:5n-3), and docosahexaenoic acid (DHA; C22:6n-3) [170]. Humans are unable to synthesize omega-3 fatty acids, and these must be obtained from the diet; ALA is mainly found in nuts and vegetable oils such as flaxseed, soybean, and canola, whereas DHA and EPA are primarily found in seafood and fish such as tuna or salmon [171]. DHA and EPA are the main omega-3 fatty acids in the CNS [172] and are known for their beneficial effects in neurodevelopment [173]. In fact, omega-3 fatty acids deficiency is associated with several nervous system disorders [174,175]. Almost all omega-3 fatty acids are located in the cell membrane layer and confer membrane fluidity at synaptic regions, which is crucial for maintaining membrane integrity and, consequently, neuronal excitability and synaptic function [176].

Omega-3 fatty acids, including ALA [177,178,179,180], EPA [181,182], and DHA [183,184,185,186], have demonstrated beneficial effects in different adult rodent models of focal ischemia [187]. Likewise, omega-3 fatty acids —especially DHA— have shown promising neuroprotective properties in several studies with neonatal rodent models of HI, both when given as maternal dietary supplementation during pregnancy [58,59,61,62,63,64] and when administered intraperitoneally to P7–P10 pups before a HI insult [45,60,65,66,67]. Several studies followed a dietary supplementation strategy in which maternal diet was enriched with omega-3 fatty acids and demonstrated that the consumption of omega-3 fatty acids during pregnancy and lactation clearly modulated the fatty acid composition in maternal milk [58] and pups’ brains [58,61,62,64]. Using the Rice–Vannucci HI rat model, a study by de Barros Mucci et al. [58], which supplemented dams with flaxseed (rich in DHA’s precursor ALA) from E1 to P21, showed that omega-3 fatty acid supplementation reduced brain damage and improved depressive behavior and spatial memory. Suganuma et al. [64] followed a similar dietary enrichment regime using fish oil and showed that DHA provided neuroprotection by inhibiting oxidative stress and apoptotic neuronal death; whereas, Decker et al. [59] supplemented dams with a menhaden fish-oil-enriched diet from E1 to P12 and, after repetitive episodes of hypoxia during the neonatal period, showed that omega-3 fatty acids prevented apoptosis and preserved striatal dopamine levels (a neurotransmitter that is very vulnerable to such insults).

Through a series of studies, Zhang et al. [61,62,63] have shed light on the neuroprotective mechanisms of DHA and EPA in neonatal HI. The authors supplemented dams from E2 to P14 using a fish-oil-enriched diet. DHA and EPA dietary supplementation reduced brain tissue loss and edema [61,62,63], inhibited the inflammatory response secondary to HI by reducing the levels of activated microglia and several neuroinflammatory markers (IL-1α, IL-1β, IL-6, COX-2, and iNOS) [61], and protected against HI-induced cell death by promoting the formation of membrane phosphatidylserine and activating the prosurvival PI3K/Akt signaling pathway [62]. DHA and EPA also preserved BBB integrity and prevented the elevation of matrix metalloproteinases [63]. The neuroprotection exerted by these omega-3 fatty acids translated into better long-term sensorimotor and cognitive outcomes [61,62], as shown by others [58].

Besides these studies that have followed a maternal dietary supplementation approach, several authors have proved the beneficial effects of omega-3 fatty acids when administered just prior to HI injury via intraperitoneal injection. Williams et al. [60] found that two doses of fish oil triglyceride emulsions (rich in DHA and EPA), administered immediately after the common carotid artery ligation and the hypoxic period, reduced brain infarct volume. Berman et al. showed that pretreatment with 1 mg/kg DHA, injected intraperitoneally 2.5 h before HI, attenuated brain damage and improved functional outcomes following neonatal HI [65], also in the context of *E. coli* lipopolysaccharide-induced systemic inflammation [66]. Arteaga et al. [67] administered the same pretreatment of 1 mg/kg DHA 10 min before HI, which reduced brain infarct volume and morphological damage, decreasing the loss of myelination, the astroglial reactive response, and microglial activation. DHA pretreatment also preserved synaptic function as well as mitochondrial inner membrane integrity and transmembrane potential. DHA not only exerted neuroprotection in the neonatal stage but also enhanced cognitive performance in adulthood, translating into better long-term memory and behavioral outcomes [67]. The same authors have also shown that DHA pretreatment can restore the auditory brainstem functional response and reduce morphological damage in the inferior colliculus [45].

Overall, there is a growing body of evidence that supports the neuroprotective properties of omega-3 fatty acids, especially of DHA [187], in reducing tissue and cell damage caused by neonatal HI and improving the neurobehavioral deficits secondary to this neuropediatric pathology. The observation that maternal diet can alter the omega-3 fatty acids content in the offspring’s brains [58,61,62,64] opens up an interesting avenue to further investigate this nutritional and transgenerational approach for the prevention of neonatal HI.

## 5. Vitamins

Vitamins are organic molecules needed for normal physiological functioning that are emerging as potential primary therapeutics [38]. Vitamins have been largely researched for their roles as essential nutrients in physiology, but recently, research has begun to examine how they are involved in nervous system dysfunction, from chronic diseases to acute insults like HI. The vitamins discussed below were selected on the basis of existing evidence demonstrating neuroprotective benefits in the prevention of neonatal HI damage.

### 5.1. Vitamin A

Vitamin A is a group of fat-soluble essential nutrients that include preformed retinoids such as retinol and its derivatives —retinal and retinoic acid— as well as a variety of provitamin A carotenoids such as β-carotene. Vitamin A plays a pivotal role in essential biological processes as a regulator of vision, reproduction, immunity, apoptosis, growth, and development. Retinoic acid (RA) is the active metabolite of vitamin A, and it exerts most of the biological effects [188]. RA interacts with two major families of nuclear receptors: retinoic acid receptors (RARs) and retinoid X receptors (RXRs). Each family is composed of three isotypes: α, β, and γ. RA can modulate the transcription of multiple downstream target genes and functional proteins through RAR-mediated signal transduction [189]. RA is involved in the regulation of the specification, patterning, and differentiation of neural stem cells in the developing mammalian nervous system [190], and RARα has been pinpointed as the main RA receptor in the hippocampus during rat neurodevelopment [191].

Using the Rice–Vannucci method in P7 rats, two studies investigated the effects of vitamin A deficiency and vitamin A supplementation on HI damage (using 300 or 7000 IU/kg/day, respectively). Maternal vitamin A deficiency (before and during the whole pregnancy and lactation) impaired the learning ability and spatial memory of pups, whereas vitamin A supplementation could alleviate these deleterious effects [68,69]. Vitamin A deficiency also aggravated hippocampal cell apoptosis induced by HI, whereas normal vitamin A levels reduced cell death by inhibiting the apoptotic caspase-3 and caspase-8/Bid pathways. Additionally, vitamin A activated the mitochondrial PI3K/Akt signaling pathway via the RARα receptor [68]. Vitamin A supplementation was also shown to increase neural stem cell proliferation in the hippocampus via RARα-mediated modulation of β-catenin signaling [69]. These results suggest that vitamin A can exert a neuroprotective effect in the context of neonatal HI by promoting neuronal survival and proliferation, which positively affects neurocognitive outcomes.

### 5.2. Vitamin B_9_

Vitamin B_9_ or folic acid is a water-soluble vitamin important for the correct development of the fetus. Folic acid is naturally present in a wide variety of foods, like beef liver and dark green leafy vegetables such as spinach and brussels sprouts. It is well known for its role in the closure of the neural tube, and, in fact, periconceptional folic acid supplementation has helped to reduce the incidence of some neural tube defects [24,192]. Folic acid also plays a key role in nucleotide biosynthesis, in the production of the universal methyl donor *S*-adenosyl-methionine (used in the methylation of DNA, histones, proteins, and lipids) [193], as well as in the remethylation of homocysteine, a cytotoxic amino acid that can induce DNA strand breakage, oxidative stress, and apoptosis [193,194]. The latter function is thought to be involved in the neuroprotection by folic acid observed in some experimental models of neurodegenerative disorders [194], CNS injury [195], and stroke patients [196].

Using the Rice–Vannucci method in P7 rats, two studies by Deniz et al. [71,197] examined the effects of maternal folic acid supplementation during pregnancy with 2 or 20 mg/kg/day (normal and excessive doses, respectively). Both folic acid doses prevented long-term HI-induced memory impairments and brain-derived neurotrophic factor imbalance in adult rats (evaluated at P60), but folic acid did not reduce hippocampal cell death [71]. These authors had previously shown that folic acid prevented memory deficits when administered to pups after an HI injury [198]. Conversely, gestational folic acid supplementation did not affect somatic growth or the early neurobehavioral development of pups (evaluated from P6 to P19). However, the high folic acid dose (20 mg/kg/day) resulted in an impairment of Na^+^,K^+^-ATPase activity in their hippocampus, an enzyme that has been correlated with memory and learning processes [197]. These results suggest that folic acid may have a dual time-dependent effect; its potential neuroprotection and its effects at an excessive dose need to be better understood during the pups’ late development.

### 5.3. Vitamin D

Vitamin D is a group of fat-soluble secosteroids, with vitamin D_3_ or cholecalciferol being the most important of these compounds in humans. The primary source of vitamin D is ultraviolet B radiation from sunlight, which penetrates the skin and activates the metabolic synthesis of cholecalciferol; however, vitamin D can also be obtained from the diet in oily fish such as salmon or tuna [199,200]. In addition to its well-established functions in bone metabolism, vitamin D has other biological activities, including anti-inflammatory, antioxidant, and antiapoptotic properties [199,201,202]. To our knowledge, no studies have yet investigated vitamin D supplementation during pregnancy and its effect on neonatal HI. In a prospective study performed in 61 neonates (30 healthy-term neonates and 31 neonates with HI), vitamin D seemed to play a neuroprotective role as its levels were significantly lower in infants with HI and their mothers. The control group presented higher levels of vitamin D as well as lower levels of oxidative stress markers and antioxidant enzyme activity [203]. These findings suggest that vitamin D may be a good neuroprotective candidate against HI damage and may reduce oxidative stress. However, further exploration of its effects in animal models of neonates and pregnant dams needs to be conducted to test this hypothesis.

### 5.4. Vitamin E

Vitamin E is a group of eight plant-derived, fat-soluble compounds that include four tocopherols and four tocotrienols (α, β, γ, and δ), α-tocopherol being the one with the highest biological activity [204]. Vitamin E was first described as a dietary factor essential for rat fertility and was soon after identified as an antioxidant of polyunsaturated lipids. Besides its potent antioxidant properties, different forms of vitamin E act as signaling and gene regulation molecules involved in inflammation, lipid homeostasis, and atherosclerotic plaque stability [204]. Clinical and preclinical studies support the beneficial effects of vitamin E in several conditions such as cardiovascular disease [205], cancer [206], or neurological disorders [207,208]. Studies of vitamin E deficiency in humans and animal models have established the critical roles of this vitamin in protecting the CNS, especially the cerebellum, from oxidative damage and motor coordination deficits [207].

In 1984, a clinical trial investigated the influence of vitamin E on the incidence of intraventricular hemorrhage (IVH) in premature infants. A total of 134 infants with birth weights ≤1500 g was recruited for the study and were given intramuscular injections (10–15 mg/kg at four time points) plus oral supplementation of vitamin E (100 mg/kg/day of α-tocopheryl acetate for 8 weeks after birth via nasogastric tube) or oral supplementation alone. Both the incidence and severity of IVH were significantly reduced in the infants who received intramuscular injections plus oral supplementation [42]. HI injury that occurs in the immediate perinatal period is thought to be a major predisposing insult leading to IVH, and the results of this study suggested that vitamin E may prevent IVH if given as soon as possible after birth to very low-birthweight infants. Moreover, in another study of vitamin E using a rat model of HI, 1.5 mg of α-tocopherol acetate was administered via subcutaneous injection to female pups at P4, P6, and P8, with HI induction at P7. Vitamin E treatment prevented the effects of HI damage on oxidative stress and inflammation markers, including the inducible and neuronal nitric oxide synthases (iNOS and nNOS) and some insulin-growth-factor-related proteins [70]. While vitamin E has high lipid solubility and low toxicity, it takes a considerable amount of time to reach effective levels in the CNS and can cause hemorrhage at very high doses [38]. These limitations should be considered when considering vitamin E as a potential neuroprotectant.

## 6. Other Neuroprotective Natural Compounds

### 6.1. Plant-Derived Compounds

In addition to polyphenols and vitamins, there are other natural products of plant origin that can protect the neonatal brain from HI insults. We summarize below the findings regarding the preventive properties of tanshinones, sulforaphane, and capsaicin; for a recent review of other plant extracts and plant-derived compounds that can be used for the treatment of neonatal HI, see [209].

#### 6.1.1. Tanshinones

Tanshinones (including cryptotanshinone, dihydrotanshinone I, tanshinone I, tanshinone IIA, and tanshinone IIB) are the main active ingredients in *Salvia miltiorrhiza* Bunge, a perennial plant native to China and Japan. Tanshinones are lipophilic diterpenoids and have the potential to penetrate the BBB [210,211,212]. The roots of *S. miltiorrhiza*, known as Danshen, are widely used in Oriental medicine for the treatment of different pathologies such as hyperlipidemia, stroke, and cardiovascular and cerebrovascular diseases [213]. Tanshinones—especially tanshinone IIA, which is one of the most abundant constituents in Danshen—have demonstrated antioxidant and anti-inflammatory benefits in the prevention of cerebral ischemic injury in animal models [214,215,216]. Beneficial results have also been reported in neonatal HI models. In relation to tanshinone I, intraperitoneal administration of 5 mg/kg/day from P6 to P12 significantly alleviated motor, memory, and spatial learning deficits in the Rice–Vannucci P7 rat model. These behavioral changes were accompanied by a significant decrease in the number of neuronal loss in the hippocampal CA1 region. Additionally, tanshinone I displayed antioxidative activity, and it significantly increased the production of glutathione peroxidase, superoxide dismutase, and catalase and reduced the production of the pro-oxidants H_2_O_2_ and iNOS [72]. In another study, Xia et al. [73] provided a daily dose of 10 mg/kg of tanshinone IIA to offspring via intraperitoneal injection from P5 to P9/P21 and induced HI following the Rice–Vannucci method at P7. Tanshinone IIA reduced the severity of brain injury, increasing ipsilateral brain weight and neuron density, and potentiated the recovery of sensorimotor functions. Compared to vehicle-treated rats, the plasma of those pups that received tanshinone IIA exhibited higher antioxidant capacity [73]. Notably, neuroprotection against neonatal HI has also been reported following tanshinone IIA post-treatment in mice [217] and rats [218].

#### 6.1.2. Sulforaphane

Sulforaphane (1-isothiocyanato-4-(methylsulfinyl)butane) is a naturally occurring organosulfur phytochemical found in cruciferous vegetables such as broccoli, Brussels sprouts, and cabbage. Sulforaphane is an isothiocyanate, plant-derived compound known for its potent antioxidant and anti-inflammatory properties; it can reduce cytotoxicity in the CNS with apparently very little toxicity [219,220] and is a known potent activator of the Nrf2 transcription factor and the Nrf2/HO-1 axis, which participate in adaptive and protective responses to oxidative stress [221,222,223]. Evidence of neuroprotective effects have been observed in rodents, in which sulforaphane was shown to reduce brain infarct volume following focal cerebral ischemia [224,225] by suppressing the inflammatory response [225,226]. Regarding neonatal HI, in P7 rat pups subjected to HI injury where sulforaphane (5 mg/kg) was administered intraperitoneally 30 min before the insult, sulforaphane pretreatment reduced brain infarct volume, apoptosis in the cortex and hippocampus, and the levels of activated microglia. This was accompanied by a reduction of caspase-3 activity and lipid peroxidation levels and an increase in the expression of Nrf2 and HO-1 in the brain [74]. Similar results were observed when sulforaphane was administered as a post-treatment 15 min after the insult in a piglet model of neonatal HI; sulforaphane increased cell viability and induced Nrf2 activation in the putamen and sensorimotor cortex [227]. These observations [74,227] suggest that sulforaphane may protect the neonatal brain against HI injury through the induction of Nrf2. The same neuroprotective effects appear to be true in the hypoxic conditions resulting from chronic placental insufficiency and subsequent intrauterine growth restriction, following a maternal dietary supplementation rich in sulforaphane, consisting of 200 mg/day of dried broccoli sprouts administered from E15 to P14 (equivalent to ~500 µg of sulforaphane per day [75]). Histological assessment revealed diminished white matter, ventricular dilation, astrogliosis, and a reduction in hippocampal neurons in injured animals compared to controls, whereas broccoli sprouts supplementation improved injured pups’ outcome in all histological assessments. This supplementation also prevented the detrimental neurocognitive effects of chronic intrauterine ischemia, such as the emergence of early reflexes or sensorimotor behaviors [75]. These results indicate that sulforaphane or sulforaphane-rich vegetables may have the potential to be used as dietary supplementation during pregnancy to protect against brain tissue damage and neurobehavioral deficits secondary to placental insufficiency.

#### 6.1.3. Capsaicin

Capsaicin (8-methyl-*N*-vanillil-6-nonenamide) is an oleoresin and is the active component of hot peppers (*Capsicum annuum* L.), one of the most common sources of spice in the Solanaceae family. Capsaicin has long been known to excite nociceptive neurons by increasing their membrane permeability to cations. Its receptor, the transient receptor potential vanilloid 1 (TRPV1), is highly expressed in spinal and peripheral nerve terminals and has an important role in nociception and analgesia. TRPV1 mediates an increase in calcium influx that promotes excitotoxic cell death mechanisms in neurons when activated [228,229]. Several studies have shown that capsaicin can also activate pathways of cell survival and decrease oxidative stress and inflammation [230,231,232]. In adult rodent models, capsaicin has been proven to provide neuroprotection against excitotoxic and ischemic brain injury through the desensitization of TRPV1 [233,234], whereas its derivative dihydrocapsaicin has also shown neuroprotective properties against transient focal ischemia in vivo [235,236,237]. There are few studies referring to neonatal models; Khatibi et al. [76] carried out research where capsaicin (0.2 or 2 mg/kg) was administered intraperitoneally to P10 rat pups 3 h before HI induction. Capsaicin pretreatment reduced brain infarct volume and improved the myogenic tone of the middle cerebral artery with either the low-dose or high-dose treatment [76]. More studies in neonatal models of HI will need to be conducted to determine the potential of capsaicin in the clinical setting.

### 6.2. Endogenous Compounds

#### 6.2.1. Melatonin

Melatonin (*N*-acetyl-5-methoxytryptamine) is a small lipophilic indoleamine produced endogenously in the pineal gland that plays a physiological role in the regulation of the sleep–wake cycle by controlling circadian rhythms. Melatonin easily crosses the BBB, and its metabolites are powerful scavengers of oxygen and nitrogen free radicals [238,239,240]. Melatonin also acts as an indirect antioxidant by increasing the efficiency of mitochondrial electron transport and by activating some of the major antioxidant enzymes, including superoxide dismutase and glutathione peroxidase [241]. Melatonin has shown neuroprotective properties against different neurological disorders such as Alzheimer’s disease [242,243], amyotrophic lateral sclerosis [244,245,246], and stroke [247,248]. With regards to neonatal HI, numerous studies have investigated melatonin as a post-treatment using animal models [249,250,251,252,253], in addition to several randomized clinical trials in humans, which support its neuroprotective role as adjuvant therapy for the treatment of HI [254,255,256] (see [257,258] for a review). The focus of this review is those studies that evaluated the neuroprotection with melatonin as a pretreatment for neonatal HI damage, either as a maternal supplementation regime [77,78] or when administered to the pups prior to HI induction [36,79,80].

Regarding maternal supplementation, Hutton et al. [77] reported that in the spiny mouse (*Acomys cahirinus*), supplementing the dams with 0.1 mg/kg/day of melatonin during the last week of gestation resulted in a reduction of CNS inflammation (macrophage infiltration and microglia) and apoptosis markers 24 h after the pups were subjected to birth asphyxia. The authors also showed that maternal melatonin easily crossed the placenta and reached fetal circulation, as has been shown for humans [259]. Another study performed in rats also showed positive results when melatonin was administered as maternal dietary supplementation during the whole pregnancy at a daily dose of 4 mg/kg/day. Pups presented less mitochondrial damage and less degeneration in pyramidal cells in the CA1 and CA3 regions of the hippocampus after uterine fetal ischemia when compared to the control group [78].

In other studies, melatonin was administered to the pups before HI damage. Using the Rice–Vannucci HI model in P7 rat pups, intraperitoneal injection of 5 or 15 mg/kg melatonin 30 min before the ischemic procedure reduced brain tissue loss [79,80] and ameliorated oxidative stress [80]. The same investigators also demonstrated that administration of three doses of 15 mg/kg melatonin after the insult (at 5 min, 24 h, and 48 h post-HI) improved long-term behavioral and learning deficits as well as brain damage in adult rats [79]. Finally, Bouslama et al. [36] showed that in a glutamate-induced excitotoxicity model of perinatal brain injury in P5 rats, a dose of 5 mg/kg melatonin administered 15 min before the damage reduced excitotoxic white-matter lesions and preserved the ability to develop conditioning. Overall, melatonin is one of the most extensively studied nutraceuticals in regard to neonatal HI. There is robust literature on its neuroprotective properties (reviewed in [258]), including several clinical trials supporting its benefits as adjuvant therapy in newborns affected by HI [254,255,256]. Preclinical studies had shown promising results when administered as prophylactic dietary supplementation during pregnancy [77,78], warranting future pilot studies to study this gestational dietary intervention in humans.

#### 6.2.2. l-Carnitine

Carnitine (3-hydroxy-4-*N*-trimethylammonium-butyrate) can be obtained in the diet from animal products like meat, fish, or milk, and it is also synthesized endogenously in the kidney, liver, and brain. Its active stereoisomer is l-carnitine, and it has an essential role in transporting and modulating potentially toxic activated long-chain fatty acids (long-chain fatty acyl-CoAs) into the mitochondria matrix for degradation by β-oxidation [260]. It has been suggested that neonates with HI suffer from carnitine deficiency [261]. In recent years, there has been considerable interest in the therapeutic potential of l-carnitine and its acetylated derivative, acetyl-l-carnitine, leading to multiple studies exploring its neuroprotective role in repairing mitochondrial function and improving functional recovery in various brain injuries [38,260]. Preclinical studies show that l-carnitine and acetyl-l-carnitine can improve energy status, decrease oxidative stress, and prevent subsequent cell death in models of adult, neonatal, and pediatric brain injury [260]. Regarding neonate models, Wainwright et al. [81,82] demonstrated that in P7 rat pups subjected to HI, pretreatment with l-carnitine (16 mmol/kg) 30 min before the injury improved brain damage. Treated pups presented less tissue loss in the ipsilateral hemisphere as well as a reduction in neuronal cell death in the cortex and hippocampus [81]. The authors proposed that l-carnitine could prevent the accumulation of acyl-CoAs in the mitochondria, which they hypothesized is a key early event involved in the pathophysiology of HI injury [82]. Using the same neonatal HI model in P7 rats, a similar reduction in apoptotic cell death in the hippocampus and the striatum (but not in the cortex) was attributed to l-carnitine pretreatment (200 mg/kg) immediately prior to HI induction [83]. However, there seems to be no consensus on the neuroprotective effects of l-carnitine when administered after the HI insult. Thus, while some researchers have found no obvious improvement [81,83], others have shown that subsequent treatment with acetyl-l-carnitine resulted in a reduction of long-term morphological and functional damage [262]. Overall, given the extensive clinical experience of l-carnitine in the treatment of pediatric cardiopathies and its minimal toxicity [263], this compound may represent an attractive candidate for neonatal HI therapy; however, more studies are warranted.

#### 6.2.3. Creatine

Creatine (2-[carbamimidoyl(methyl)amino]acetic acid) is a guanidine compound found in fish and meat that is also synthesized endogenously from arginine, glycine, and *S*-adenosylmethionine. Creatine is an essential compound for cellular energy metabolism homeostasis, and its phosphorylated form (phosphocreatine) is the source of phosphate in the conversion of ADP to ATP [264]. Creatine ameliorates oxidative stress, glutamatergic excitotoxicity, and apoptosis in vitro and in vivo [265], and, as a supplement, it has been shown to increase both creatine and phosphocreatine levels in the brain, providing functional benefits in a great number of experimental models of neurological disease [266,267]. For instance, creatine supplementation during pregnancy was shown to positively affect the morphological and electrophysiological development of hippocampal neurons in offspring rats, increasing neuronal excitability [268]. These positive effects were maintained in adult rats, which retained enhanced neuron excitability and long-term potentiation [269]. Creatine administered during pregnancy was shown to cross the placenta and reach several organs of the fetus, and, in fact, maternal creatine supplementation has been proposed as prophylaxis to protect the fetus from the multiorgan consequences of severe hypoxia at birth [270]. In the spiny mouse, maternal creatine administered from midpregnancy increased creatine levels in the fetal brain and the ability of offspring to survive an episode of acute birth asphyxia [84], protecting the brain from perinatal hypoxia by reducing lipid peroxidation and apoptosis and preserving mitochondrial function [85]. In a neonatal rat model of HI, pups received subcutaneous injections of 3 g/kg/day of creatine monohydrate for 3 consecutive days (P6–P8), with HI induction at P7. Creatine supplementation significantly increased the energy potential (i.e., the levels of phosphocreatine in the brain, measured at P9), and it showed a 25% reduction in brain edema compared with controls [86]. A similar regime of creatine supplementation (subcutaneous injection of 3 g/kg at four time points, three before HI and the latest 3 h after the injury) in rat pups that underwent HI at P7 resulted in a reduction of brain injury severity and cell loss in the cortex and hippocampus [87]. Moreover, several studies have shown that creatine supplementation after an HI event is neuroprotective, reducing neuroinflammation and improving cognitive and motor functions [271,272,273]. In the light of the positive results available (for a review, see [270]), it would be interesting to further investigate whether maternal creatine supplementation may protect the fetal brain from neonatal HI.

#### 6.2.4. Lactate

Lactate (2-hydroxypropanoate), an intracellular metabolite of glucose, is the anion resulting from the dissociation of lactic acid. Lactate is a source of metabolic energy regarded as an important supplementary fuel for neurons since the healthy brain uses lactate rather than glucose as an efficient energy substrate to maintain synaptic transmission [274], whereas the immature brain has a high capacity to use lactate as an energy substrate [275]. l-Lactate, both after intracerebroventricular and intravenous administration, protects neurons under pathologic conditions, and it has been proven to be neuroprotective against excitotoxicity in several preclinical models of acute brain injury, including ischemia, intracerebral hemorrhage, and traumatic brain injury [276,277,278]. Regarding neonatal HI, a single intraperitoneal injection of sodium lactate (2.14 mmol/kg) to P7 rats immediately before hypoxia resulted in a 30% reduction in brain lesion volume, whereas after three injections of lactate post-HI, the pups had the lowest brain lesion volume and no differences in neurological reflexes, sensorimotor abilities, and long-term memory compared to controls [88]. Similar neuroprotection against brain lesions and behavioral deficits were also reported by other groups when l-lactate was administered after a HI insult in neonatal rats [279]. Based on these initial promising results, it would be appropriate to further explore whether l-lactate pretreatment could play a role in the prevention of neonatal HI.

## 7. Conclusions and Future Perspectives

This review summarizes the current knowledge on natural products that can prevent the brain damage and functional impairments consequences of neonatal HI encephalopathy when administered before the damage occurs. These nutraceuticals include polyphenols, omega-3 fatty acids, vitamins, and several other plant-derived and endogenous compounds. They share some common characteristics: most of them are obtained from the diet or are the main active components in some traditional Oriental medicines, and they have known antiapoptotic, antioxidant, and anti-inflammatory properties, among others. Hence, the compounds herein reviewed are thought to exert their neuroprotection against neonatal HI via multiple mechanisms, which have been summarized in Table 2. The most common mechanisms involved in neuroprotection are (1) the reduction of neuronal cell death by modulating the apoptotic cascade and activating prosurvival signaling pathways; (2) the reduction of oxidative stress by modulating enzymatic activity, reducing ROS levels, and preserving mitochondrial inner membrane integrity and transmembrane potential, and (3) the reduction of the neuroinflammatory response by ameliorating astrogliosis and microgliosis and decreasing the levels of proinflammatory cytokines. All these processes —cell death [8], oxidative stress [10], and neuroinflammation [13]— are known to contribute to different stages of HI pathophysiology [6,7]. Moreover, several of the studies reviewed identified two signaling pathways that mediated the nutraceuticals’ neuroprotection: the PI3K/Akt signaling pathway [53,56,62,68], a prosurvival pathway that regulates various processes, including cell growth, apoptosis or glucose metabolism, and the Nrf2/HO-1 signaling pathway [46,50,74], which regulates mitochondrial oxidative stress and calcium homeostasis. These intracellular cascades are also known to confer neuroprotection against ischemic stroke [224,280], and, therefore, they represent interesting targets for the development and evaluation of future therapeutic interventions against HI.

Some of the natural products reviewed have been extensively studied for the prevention and treatment of ischemic stroke using adult rodent models. Although there are differences between neonatal and adult brains, ischemic stroke and HI share some common pathophysiological features, and, therefore, these studies provide additional supportive evidence of the neuroprotective potential of nutraceuticals such as resveratrol [281,282,283,284], quercetin [127,128,129,130], mangiferin [141,142,143], pomegranate juice polyphenols [147,148], omega-3 fatty acids [177,178,179,180,181,182,183,184,185,186], tanshinones [214,215,216], sulforaphane [224,225,226], and capsaicin and its derivatives [233,234,235,236,237]. Moreover, there are other natural compounds with antioxidant and anti-inflammatory activities —not yet examined in the context of neonatal HI— that have shown to be neuroprotective against ischemic brain injury. That is the case of olive oil phenols [285], a major constituent of the Mediterranean diet, the consumption of which has been inversely correlated with the incidence of ischemic cerebrovascular disease [286]. Dietary supplementation with olive oil or some of its phenolic compounds can ameliorate brain damage and neurological deficits secondary to focal ischemia in vivo [287,288,289,290]. Given the positive results observed using olive oil phenols in reducing ischemic damage in the adult brain, it would be interesting to investigate the potential of this nutraceutical against neonatal HI.

Of special interest are those natural products that can be administered as dietary supplements to the mothers during pregnancy and/or lactation to protect the fetal and neonatal brain from HI injury. Many brain injuries have their onset in utero [1], and diet, gut microbiome (which affects the metabolization and absorption of macro- and micronutrients and determines the composition of the fetus microbiota), and other environmental factors during early development are known contributors to lifelong disease patterns [291,292,293]. Thus, pregnancy may represent a therapeutic window for intervention, and several authors have reviewed and discussed the benefits of maternal dietary interventions as prophylaxis for perinatal brain injury, including HI encephalopathy [1,270,294].

Current preclinical evidence (Table 1) supports the neuroprotective benefits of maternal supplementation using omega-3 fatty acids [58,59,61,62,63,64], piceatannol and resveratrol [47,48,49], vitamin A [68,69], melatonin [77,78], or creatine [84,85], as well as dietary interventions consisting of pomegranate juice (rich in polyphenols) [41,43,54] or broccoli sprouts (rich in sulforaphane) [75]. The advantage of using nutraceuticals (over traditional drugs) for HI prevention is that these compounds usually have low toxicity, have minimal interactions with other drugs, and are comparatively inexpensive and broadly accessible [38,39,40]. However, most of the aforementioned studies did not examine embryotoxicity or any potential detrimental effects to the mothers (or, at least, these were not reported). The safety profile in pregnant mothers and embryos would need to be carefully evaluated before translating their use into humans, especially for those interventions designed as dietary supplements to be taken during pregnancy. Moreover, the Rice–Vannucci method with injury induction at P7 (comparable to 36–40 weeks of gestation in humans [25]) is, to date, the most widely used model for the study of neonatal HI. Still, the mode and timing of the nutritional intervention administration varied a lot between the studies (Table 1). It would be beneficial to standardize supplementation protocols to allow cross-study comparisons in order to foster the advancements in this field.

To our knowledge, there have only been two human studies that have examined the preventive effects of nutraceuticals as prophylaxis for HI-related pathologies. One clinical trial evaluated the effects of vitamin E administration to very low-birthweight infants on the prevalence and severity of IVH [42], and a recent pilot study examined the benefits of pomegranate juice intake on pregnancies affected by intrauterine growth restriction [41]. Fear of harming the developing fetus remains due to errors of the past, such as the release of thalidomide. However, simple nutritional interventions, including maternal supplementation with folic acid or iron, have been successful in reducing the risk of neural tube defects, anemia, and low birthweight [23,24,295]. Given the inherent difficulty of predicting the occurrence of neonatal HI, treatments that prevent, rather than rescue, perinatal brain injury are likely to be the most effective, especially for high-risk pregnancies (e.g., those affected by intrauterine growth restriction or placental insufficiency) as well as in low-resource settings. It would, therefore, be convenient to conduct more human studies to test gestational nutritional interventions in the prevention of HI, especially for those nutraceuticals (such as omega-3 fatty acids or melatonin) that are supported by an extensive body of preclinical evidence.

To conclude, neonatal HI is a limiting pathology that affects a young population and is often associated with lifelong mental and physical disabilities. Given the limited therapies currently available to treat neonates suffering from HI once the damage has occurred, nutritional interventions may provide the ideal platform for therapies that can be administered safely and prophylactically to prevent and reduce the brain damage and neurological impairments consequences of HI encephalopathy in newborn babies. We hope that this review will highlight the importance of natural product interventions during pregnancy and lactation and encourage a much-needed preclinical and clinical research in this field.

## Figures and Tables

**Table 1:** Summary of the preclinical in vivo studies that have examined the neuroprotective effects of natural products administered before neonatal hypoxia–ischemia (HI) injury.

Compound	Organism	HI Injury Method *	Injury Date	Administration Timing	Administration Duration ^¶^	Dose	Administration Mode	Neuroprotective Effects	Ref
***POLYPHENOLS***									
Resveratrol	Mouse and rat	RV: 8% O_2_ for 45 min (mouse) or 2.5 h (rat)	P7	Pups: 24 h or 10 min before H	Single dose	0.002, 0.2 or 20 mg/kg	i.p. injection	↓ Tissue loss in hippocampus and striatum, ↓ apoptotic and necrotic cell death	[43]
	Rat	RV: 8% O_2_ for 2 h 15 min	P7	Pups: 10 min before H	Single dose	20 mg/kg	i.p. injection	↓ Brain infarct volume, loss of myelination and cell loss in cortex and hippocampus, ↓ ROS production, ↑ maintenance of the mitochondrial inner membrane integrity and transmembrane potential, ↓ long-term cognitive impairments and functional damage	[44]
	Rat	RV: 8% O_2_ for 2 h 15 min	P7	Pups: 10 min before HI	Single dose	20 mg/kg	i.p. injection	↓ Morphological damage and astrogliosis in the inferior colliculus, ↓ loss of myelination, restored the auditory brainstem functional response	[45]
	Rat	RV: 8% O_2_ for 2.5 h	P14	Pups: P7–P14	7 days	20 or 40 mg/kg/day	i.p. injection	↓ Brain infarct volume, ↓ cerebral edema, ↓ neuroinflammation, ↓ oxidative stress, ↑ Nrf2/HO-1 signaling pathway	[46]
	Rat	Helmy model: 9% O_2_ + 20% CO_2_ for 90 min	P6	Mothers: from end of weaning till pups reached P7	88–98 days ^†^	50 mg/kg/day	Drinking water	↓ Neuroinflammation in hippocampus	[47]
	Rat	RV: 8% O_2_ for 2 h	P7	Mothers: E15–P9	15 days	0.15 mg/kg/day	Drinking water	Partially ↓ sensorimotor defects and long-term memory deficits in the context of moderate maternal alcohol consumption	[48]
Piceatannol	Rat	RV: 8% O_2_ for 2 h	P7	Mothers: E15–P7 or P0–P7	6 or 14 days	0.15 mg/kg/day	Drinking water	↓ Brain infarct volume and anatomical brain lesions, ↓ cerebral edema, ↓ neuronal apoptosis, ↑ early reflexes, ↓ sensorimotor defects and long-term cognitive impairments	[49]
	Rat	RV: 8% O_2_ for 2 h	P7	Mothers: E15–P9	15 days	0.15 mg/kg/day	Drinking water	↓ Sensorimotor defects and cognitive impairments in the context of moderate maternal alcohol consumption	[48]
Pterostilbene	Rat	RV: 8% O_2_ for 2 h	P7	Pups: 30 min before HI	Single dose	50 mg/kg	i.p. injection	↓ Brain infarct volume, ↓ brain edema, ↓ neuronal apoptosis, ↓ neuroinflammation, ↓ oxidative stress through ↑ HO-1 expression, ↓ motor and memory deficits	[50]
Quercetin	Piglet	Transient bilateral carotid ligation + 8% O_2_ for 40 min	P2	Pups: 1 h before HI	Single dose	10 mg/kg	Nanosomes injected intravenously	Improved electroencephalographic amplitude records and neurological functions, restored blood pressure and spontaneous breathing	[51]
	Rat	RV: 8% O_2_ for 2.5 h	P7	Pups: P0–P7	7 days	40 mg/kg/day	Intragastric	↓ Cortical cell apoptosis, microgliosis, and astrogliosis, ↓ neuroinflammation, ↓ TLR4/NF-κB signaling	[52]
Mangiferin	Rat	RV: 8% O_2_ for 2 h	P10	Pups: P3–P12	9 days	50, 100 or 200 mg/kg/day	Oral gavage	↓ Brain infarct volume, ↓ neuronal apoptosis, ↓ oxidative stress, ↑ PI3K/Akt signaling pathway, ↑ isoflurane’s neuroprotection	[53]
Pomegranate juice	Mouse	RV: 8% O_2_ for 45 min	P7	Mothers: E14–P8 or E14–P14	15 or 21 days	8, 16, or 32 µmol/day	Drinking water	↓ Tissue loss in the hippocampus, cortex, and striatum in a dose-dependent manner, ↓ neuronal apoptosis	[54]
	Mouse and rat	RV: 8% O_2_ for 45 min (mouse) or 2.5 h (rat)	P7	Mothers: E0–P8	29 days	4.8 mg/day	Drinking water	↓ Neuronal apoptosis	[43]
GSPE	Mouse	RV: 8% O_2_ for 2 h	P7	Pups: 20 min before HI	Single dose	30 mg/kg	i.p. injection	↓ Brain infarct volume, ↓ neuronal apoptosis, improved neurobehavioral outcomes	[55]
Icariin	Mouse	RV: 8% O_2_ for 2 h	P7	Pups: 20 min before HI	Single dose	10 mg/kg	i.p. injection	↓ Brain infarct volume, ↓ neuronal apoptosis, improved neurobehavioral outcomes, ↑ PI3K/Akt signaling pathway	[56]
Daphnetin	Rat	RV: 8% O_2_ for 2.5 h	P7	Pups: 1 h before H	Single dose	10 mg/kg	i.p. injection	↓ Brain infarct volume	[57]
***OMEGA-3 FATTY ACIDS***									
n-3 PUFAs	Rat	RV: 8% O_2_ for 90 min	P7	Mothers: E1–P21	41 days	46% *w*/*w* total fatty acids	Flaxseed-enriched diet	↓ Brain tissue loss, ↑ hippocampal n-3 PUFAs content in pups’ brains, ↓ depressive behavior and cognitive impairments	[58]
	Rat	3 hypoxic insults per day with 10% O_2_ + 3% CO_2_ for 2 h	P7–P12	Mothers: E1–P12	32 days	3.5–4 g/day	Fish-oil-enriched diet	↓ Hippocampal apoptosis, preserved striatal dopamine levels	[59]
	Mouse	RV: 8% O_2_ for 15 min	P10	Pups: postsurgery and post-H	2 doses	3 mg/day	i.p. injection	↓ Brain infarct volume	[60]
DHA and EPA	Rat	RV: 8% O_2_ for 2.5 h	P7	Mothers: E2–P14	33 days	1.5% *w*/*w*	Fish-oil-enriched diet	↓ Brain tissue loss, ↑ cortical content of DHA and EPA in pups’ brain, ↓ microgliosis, ↓ neuroinflammation, ↓ long-term sensorimotor and cognitive impairments	[61]
	Rat	RV: 8% O_2_ for 2.5 h	P7	Mothers: E2–P14	33 days	Not specified	Fish-oil-enriched diet	↓ Brain tissue loss, ↓ cortical apoptosis, ↑ phosphatidylserine, DHA and EPA content in pups’ brain, ↑ PI3K/Akt signaling pathway, improved neurological outcomes	[62]
	Rat	RV: 8% O_2_ for 2.5 h	P7	Mothers: E2–P14	33 days	1.5% *w*/*w*	Fish-oil-enriched diet	↓ Brain edema, ↑ BBB integrity, ↓ matrix metalloproteinase activity	[63]
DHA	Rat	RV: 8% O_2_ for 90 min	P7	Mothers: E7 till pups’ sacrifice at P8–P14	22–28 days	10% *w*/*w* total fatty acids	Fish-oil-enriched diet	↑ DHA content in pups’ brain, ↓ hippocampal apoptosis, ↓ oxidative stress	[64]
	Rat	RV: 8% O_2_ for 90 min	P7	Pups: 2.5 h before HI	Single dose	1, 2.5 or 5 mg/kg	i.p. injection	↓ Hippocampal tissue loss, improved functional outcomes	[65]
	Rat	RV: 8% O_2_ for 90 min	P7	Pups: 2.5 h before HI	Single dose	1 mg/kg	i.p. injection	Improved functional outcomes in the context of *E. coli* lipopolysaccharide-induced systemic inflammation	[66]
	Rat	RV: 8% O_2_ for 2 h 15 min	P7	Pups: 10 min before HI	Single dose	1 mg/kg	i.p. injection	↓ Brain infarct volume, ↓ loss of myelination, ↓ astrogliosis and microgliosis, ↑ maintenance of the mitochondrial inner membrane integrity and transmembrane potential, ↓ long-term behavioral and cognitive impairments	[67]
	Rat	RV: 8% O_2_ for 2 h 15 min	P7	Pups: 10 min before HI	Single dose	1 mg/kg	i.p. injection	↓ Astrogliosis, ↓ loss of myelination, restored the auditory brainstem functional response	[45]
***VITAMINS***									
Vitamin A	Rat	RV: 8% O_2_ for 2.5 h	P7	Mothers: 4 weeks before pregnancy till pups’ sacrifice	Whole pregnancy and lactation	300 or 7000 IU/kg/day	Diet	↓ Hippocampal cell apoptosis by ↑ caspase-3 and caspase-8/Bid pathways, ↑ PI3K/Akt signaling pathway by binding to RARα, improved learning ability, and spatial memory impairments	[68]
	Rat	RV: 8% O_2_ for 2.5 h	P7	Mothers: 4 weeks before pregnancy till pups’ sacrifice	Whole pregnancy and lactation	300 or 7000 IU/kg/day	Diet	↑ Neurol stem cell proliferation by ↑ RARα-mediated modulation of β-catenin signaling, improved learning ability, and spatial memory impairments	[69]
Vitamin E	Rat	RV: 8% O_2_ for 90 min	P7	Pups: P4, P6 and P8	3 doses	1.5 mg/day	s.c. injection	↓ Expression of iNOS, nNOS and IGF-related proteins	[70]
Folic acid	Rat	RV: 8% O_2_ for 90 min	P7	Mothers: E0 till birth	Whole pregnancy	2 or 20 mg/kg/day	Diet	↓ Long-term memory impairments, ↓ brain-derived neurotrophic factor imbalance	[71]
***PLANT-DERIVED COMPOUNDS***									
Tanshinone I	Rat	RV: 8% O_2_ for 2.5 h	P7	Pups: P6–P12	6 days	5 mg/kg/day	i.p. injection	↓ Neuronal loss in the hippocampus, ↓ motor and cognitive deficits, ↓ oxidative stress	[72]
Tanshinone IIA	Rat	RV: 8% O_2_ for 2 h	P7	Pups: P5–14 or P5–21	9 or 16 days	10 mg/kg/day	i.p. injection	↓ Brain injury, ↓ cortical cell loss, ↑ plasma antioxidant capacity, improved sensorimotor function	[73]
Sulforaphane	Rat	RV: 8% O_2_ for 90 min	P7	Pups: 30 min before HI	Single dose	5 mg/kg	i.p. injection	↓ Brain infarct volume, ↓ apoptosis in cortex and hippocampus, ↓ microgliosis, ↓ lipid peroxidation, ↑ Nrf2/HO-1 signaling pathway	[74]
	Rat	Wigglesworth model: bilateral uterine artery ligation	E20	Mothers: E15–P14	20 days	200 mg/day	Diet (dried broccoli sprouts)	↓ White matter loss and ventricular dilation, ↓ hippocampal cell loss, ↓ loss of myelination, ↓ astrogliosis, improved neurobehavioral outcomes	[75]
Capsaicin	Rat	RV: 8% O_2_ for 2 h	P10	Pups: 3 h before HI	Single dose	0.2 or 2 mg/kg	i.p. injection	↓ Brain infarct volume, ↑ the middle cerebral artery myogenic tone	[76]
***ENDOGENOUS COMPOUNDS***									
Melatonin	Spiny mouse	Bjelke model: 7.5 min intrauterine ischemia	E37	Mothers: E29–E37	8 days	0.1 mg/kg/day	Subscapula osmotic pump	↓ Macrophage infiltration, ↓ microgliosis, ↓ apoptosis in cortex	[77]
	Rat	Bilateral utero-ovarian artery occlusion for 30 min	E16	Mothers: E0 till birth	Whole pregnancy	4 mg/kg/day	Drinking water	↓ Mitochondrial damage, ↓ degeneration of pyramidal cells in the hippocampus	[78]
	Rat	RV: 8% O_2_ for 2.5 h	P7	Pups: 30 min before HI	Single dose	5 or 15 mg/kg	i.p. injection	↓ Brain tissue loss in a concentration-dependent manner	[79]
	Rat	RV: 8% O_2_ for 2.5 h	P7	Pups: 30 min before HI	Single dose	15 mg/kg	i.p. injection	↓ Brain damage in hippocampus and cortex, ↓ oxidative stress by ↓ the levels of free iron, F_2_-isoprostanes, and F_4_-neuroprostanes	[80]
	Rat	Excitotoxity injury by ibotenate injection	P5	Pups: 15 min before ibotenate injection	Single dose	5 mg/kg	i.p. injection	↓ White matter lesions, preserved the ability to develop conditioning	[36]
Carnitine	Rat	RV: 8% O_2_ for 70 min	P7	Pups: 30 min before H	Single dose	16 mmol/kg	i.p. injection	↓ Brain injury, ↓ apoptosis in hippocampus and cortex	[81]
	Rat	RV: 8% O_2_ for 70 min	P7	Pups: 30 min before H	Single dose	16 mmol/kg	i.p. injection	↓ Accumulation of acyl-CoA esters in the brain, ↓ superoxide levels, ↓ mitochondrial injury	[82]
	Rat	RV: 8% O_2_ for 1 h	P7	Pups: just before HI	Single dose	200 mg/kg	i.p. injection	↓ Apoptosis in hippocampus and striatum	[83]
Creatine	Spiny mouse	Bjelke model: 7.5 min intrauterine ischemia	E37 or E38	Mothers: E20 till sacrifice at E37–E38	17–18 days	5% *w*/*w*	Enriched diet	↑ Capacity of the offspring to survive birth asphyxia, improved postnatal growth, ↑ creatine content in brain and other fetal tissues	[84]
	Spiny mouse	Bjelke model: 7.5 min intrauterine ischemia	E38	Mothers: E20–E38	18 days	5% *w*/*w*	Enriched diet	↓ Lipid peroxidation, ↓ apoptosis, ↓ mitochondrial damage	[85]
	Rat	RV: 8% O_2_ for 100 min	P7	Pups: P6–P8	3 doses	3 g/kg/day	s.c. injection	↓ Brain edema, ↑ energy potential and the levels of phosphocreatine in the brain	[86]
	Rat	RV: 8% O_2_ for 80 min	P7	Pups: P4–P7	4 doses	3 g/kg/day	s.c. injection	↓ Brain injury, ↓ neuronal cell damage in the cortex and hippocampus	[87]
Lactate	Rat	RV: 8% O_2_ for 2 h	P7	Pups: just before H	Single dose	2.14 mmol/kg	i.p. injection	↓ Brain infarct volume	[88]

Abbreviations: GSPE, grape seed proanthocyanidin extract; i.p., intraperitoneal; n-3 PUFAs, omega-3 polyunsaturated fatty acids; RV, Rice–Vannucci; s.c., subcutaneous. * HI methods: Rice–Vannucci model of unilateral common carotid artery ligation, followed by a period of hypoxia with 8% O_2_ [30]; Helmy model of perinatal asphyxia, a combination of hypercapnia (20% CO_2_) and hypoxia (9% O_2_) [89]; Bjelke model of intrauterine ischemia or “delayed cesarean section” [35], later adapted in [84]; Wigglesworth model of chronic placental insufficiency induction by bilateral uterine artery ligation [90]; Ikonomidou excitotoxity model by ibotenate injection [37]. ^¶^ The duration of nutraceutical administration was calculated based on an average gestation length of 21 days in rats. ^†^ In [47], supplementation was initiated when female rats finished weaning (at P30) and continued while dams reached maturity for mating (at P90–P100), during the whole pregnancy, and the first week of lactation, adding up to a total of 88–98 days.

**Table 2:** Possible neuroprotective mechanisms of natural products associated with benefits against neonatal HI damage.

	Cell Death	Oxidative Stress	Neuroinflammation	BBB Integrity and Edema	Excito Toxicity	Energy Metabolism	Synaptic Function and Neuroplasticity
***POLYPHENOLS***							
Resveratrol	**x**	**x**	**x**	**x**			**x**
Piceatannol	**x**			**x**			
Pterostilbene	**x**	**x**	**x**	**x**			
Quercetin	**x**		**x**				**x**
Mangiferin	**x**	**x**					
Pomegranate polyphenols	**x**	**?**					
GSPE	**x**						
Icariin	**x**						
Daphnetin		**?**					
***OMEGA-3 FATTY ACIDS***							
EPA	**x**		**x**	**x**			
DHA	**x**	**x**	**x**	**x**			**x**
***VITAMINS***							
Vitamin A	**x**						**x**
Vitamin B_9_							**?**
Vitamin D		**?**					
Vitamin E		**x**					
***PLANT-DERIVED COMPOUNDS***							
Tanshinones	**x**	**x**					
Sulforaphane	**x**	**x**	**x**				
Capsaicin					**?**		
***ENDOGENOUS COMPOUNDS***							
Melatonin	**x**	**x**	**x**				
Carnitine	**x**	**x**				**x**	
Creatine	**x**	**x**		**x**		**x**	
Lactate		**?**				**?**	

Those marked with a question mark (?) indicate possible mechanisms suggested by the authors (see main text) that remain to be experimentally validated in preclinical models of neonatal HI.

## Abbreviations

ADNES	Advanced neuroprotection strategy
ALA	Alpha linoleic acid
BBB	Blood–brain barrier
CNS	Central nervous system
DHA	Docosahexaenoic acid
E	Embryonic day
EPA	Eicosapentaenoic acid
GSPE	Grape seed proanthocyanidin extract
HI	Hypoxia–ischemia
i.p.	Intraperitoneal
IL	Interleukin
iNOS	Inducible nitric oxide synthase
IVH	Intraventricular hemorrhage
n-3 PUFAs	Omega-3 polyunsaturated fatty acids
NO	Nitric oxide
Nrf/HO-1	Nuclear factor erythroid 2 related factor 2/heme oxygenase 1
P	Postnatal day
PI3K/Akt	Phosphoinositide 3-kinase/protein kinase B
RA	Retinoic acid
RAR	Retinoic acid receptor
ROS	Reactive oxygen species
RXR	Retinoic acid X receptor
TLR4/NF-κB	Toll-like receptor 4/nuclear factor κB
TNF-α	Tumor necrosis factor α
TRPV1	Transient receptor potential vanilloid 1
